# Impaired Oxygenation of the Prefrontal Cortex During Verbal Fluency Task in Young Adults With Major Depressive Disorder and Suicidality: A Functional Near-Infrared Spectroscopy Study

**DOI:** 10.3389/fpsyt.2022.915425

**Published:** 2022-06-23

**Authors:** Hyewon Kim, JongKwan Choi, Bumseok Jeong, Maurizio Fava, David Mischoulon, Mi Jin Park, Hyun Soo Kim, Hong Jin Jeon

**Affiliations:** ^1^Department of Psychiatry, Hanyang University Hospital, Seoul, South Korea; ^2^OBELAB Inc., Seoul, South Korea; ^3^Graduate School of Medical Science and Engineering, Korea Advanced Institute of Science and Technology, Daejeon, South Korea; ^4^Depression Clinical and Research Program, Massachusetts General Hospital and Harvard Medical School, Boston, MA, United States; ^5^Department of Psychiatry, Seoul St. Mary’s Hospital, College of Medicine, The Catholic University of Korea, Seoul, South Korea; ^6^Department of Psychiatry, Dong-A University Hospital, Busan, South Korea; ^7^Department of Psychiatry, Depression Center, Samsung Medical Center, Sungkyunkwan University School of Medicine, Seoul, South Korea; ^8^Department of Health Sciences and Technology, Department of Medical Device Management and Research, and Department of Clinical Research Design and Evaluation, Samsung Advanced Institute for Health Sciences and Technology (SAIHST), Sungkyunkwan University, Seoul, South Korea; ^9^Department of Medical Device Management and Research, Samsung Advanced Institute for Health Sciences and Technology, Sungkyunkwan University, Seoul, South Korea; ^10^Department of Clinical Research Design and Evaluation, Samsung Advanced Institute for Health Sciences and Technology, Sungkyunkwan University, Seoul, South Korea

**Keywords:** major depressive disorder, fNIRS, prefrontal cortex, oxygenated hemoglobin, suicide

## Abstract

**Background:**

Few previous studies have focused on prefrontal activation in young adults diagnosed with major depressive disorder (MDD) and suicidality *via* functional near-infrared spectroscopy (fNIRS).

**Materials and Methods:**

A total of 59 healthy controls (HCs), 35 patients with MDD but without suicidality, and 25 patients with MDD and suicidality, between the ages of 18–34 years, were enrolled. Changes in oxygenated hemoglobin (oxy-Hb) levels of the prefrontal cortex at baseline, 4 weeks, and 8 weeks, were evaluated using a protocol consisting of three consecutively repeated trials of rest, speech, and verbal fluency test (VFT) *via* fNIRS. MDD was diagnosed and suicidality was evaluated based on Mini International Neuropsychiatric Interview (MINI).

**Results:**

Oxy-Hb levels were impaired in patients with MDD compared with HCs (*p* = 0.018 for left prefrontal cortex; *p* = 0.021 for right ventromedial prefrontal cortex; *p* = 0.002 for left frontopolar cortex). Among the three groups including HCs, MDD without suicidality, and MDD with suicidality, prefrontal oxygenation was most decreased in MDD patients with suicidality. A significantly impaired prefrontal oxygenation in the right ventrolateral prefrontal cortex (VLPFC) was detected after adjusting for covariates in MDD patients with suicidality, compared to those without suicidality.

**Conclusion:**

Impaired prefrontal oxygenation during cognitive execution may serve as a diagnostic biomarker for suicidality in young adult patients with MDD.

## Introduction

Major depressive disorder (MDD) is a common psychiatric illness associated with significant morbidity (1). Symptoms of MDD are often distinguished as two symptom clusters: cognitive/affective subtype (e.g., lack of interest, suicidal thoughts, and guilt) and somatic/affective subtype (e.g., fatigue, psychomotor agitation/retardation, sleep problems, and appetite/weight disturbance) (2, 3). Previous studies have suggested that etiology or prognosis may differ according to the symptom clusters (3, 4). Suicidality, one of the cognitive/affective symptoms of MDD, is often the most immediate clinical concern among people with MDD. It is estimated that about 50% of all suicide occur within a depressive episode and people with MDD are about 20-fold more likely to die by suicide compared to the general population (5, 6).

Although the symptoms of people with MDD are highly heterogenous, age at onset is one of the most critical factors that lead to a rather homogenous subgrouping among them. The onset of MDD occurs before age of 40 years in about 50 percent of patients (7). There have been suggested that etiology, manifestation, and course of depression differ according to the age of onset; compared to later onset, earlier age at onset of MDD is associated with higher genetic loading, atypical symptoms, more irritability and anxiety, personality disorder, alcohol abuse, higher possibility of conversion to bipolar disorder, and poorer long-term outcome (7–9). Moreover, previous studies have consistently reported an increased suicidal risk in those with early onset (10–12). These findings suggest that there may be underlying neurobiological differences according to subgroup according to age.

Functional near-infrared spectroscopy (fNIRS) is an optic neuroimaging technology that uses near-infrared light to measure the local concentration of cortical oxygenated and deoxygenated hemoglobin. The fNIRS can be used to detect blood oxygen saturation by irradiating the cerebrum with near-infrared rays between a spectral window of 650 and 1000 nm and measuring the returned light with sensors according to the light absorption rate (13). Because fNIRS enables non-invasive monitoring of brain activity in real-time with similar spatial resolution and relatively higher temporal resolution compared with functional magnetic resonance imaging (fMRI) and positron emission tomography (PET), several studies have used fNIRS in the fields of psychiatry and neuroscience that utilize changes in brain activity as biomarkers (14).

Previous fNIRS studies conducted among patients with MDD have shown a reduced increase in the oxygenated hemoglobin (oxy-Hb) levels during verbal fluency test (VFT) in the prefrontal regions (15, 16) or frontotemporal regions (17, 18). In addition, the oxy-Hb concentration was negatively correlated with the degree of depression (19), and the correlation was stronger in the recurrent MDD group (20). Moreover, previous studies have shown the association of prefrontal hemodynamic changes *via* fNIRS and suicidality in patients with MDD, reporting a reduced prefrontal activation during cognitive tasks compared to those without suicidality (21, 22). Different brain regions were associated with such changes; an fNIRS study with the mean age of 37.6 years (SD, 10.0 years) and 38.8 years (SD, 9.7 years) for suicide attempters and non-attempters, respectively, showed that suicide attempters had reduced hemodynamic changes in the left precentral gyrus compared with non-attempters (22); another study with the mean age of 57.3 years (SD, 15.7 years) and 58.7 years (SD, 16.5 years) for MDD patients with suicide ideation and MDD patients without suicidal ideation, respectively, showed that the degree of suicidal ideation was negatively associated with the changes in oxy-Hb in the right orbitofrontal cortex (OFC), frontopolar cortex (FPC), and dorsolateral prefrontal cortex (DLPFC) (21); and, another fNIRS study with the mean age of 37.6 years (SD, 14.36 years) and 33.4 years (SD, 12.6 years) for patients with MDD and healthy controls (HCs), respectively, reported relatively greater left prefrontal activity during cognitive tasks in patients who diagnosed with MDD and suicidal ideation compared to those without suicidal ideation, and demonstrated that this prefrontal asymmetry (greater oxy-Hb in the left hemisphere) has a moderating effect between depression and suicidal ideation (23).

Although previous fNIRS studies have discovered features of hemodynamic changes during a cognitive task in people with MDD and suicidality, evidence in a targeted population of young adults is not yet available. In this study, we investigated hemodynamic changes in the prefrontal cortex *via* fNIRS monitoring of young adult patients with MDD. We hypothesized that (1) the increase in prefrontal cortical activity during a cognitive task is smaller in young adult patients with MDD than in HCs, and (2) differences in prefrontal hemodynamic changes occur during a cognitive task involving MDD patients with and without suicidality.

## Materials and Methods

### Participants

The study enrolled 35 patients with MDD but no suicidality, 25 patients with MDD and suicidality, and 59 healthy controls (HCs), between the ages of 18 and 34 years. We did not stratify according to sex in the recruitment of subjects, and the number of males in each group was 27, 16, and 27, respectively. Patients with MDD were recruited through the outpatient clinic of Samsung Medical Center in Seoul, South Korea, and HCs were recruited from the community through advertisements released by the Clinical Trial Center of Samsung Medical Center between July 2018 and October 2020. The native language of all participants was Korean. Diagnosis of MDD and evaluation of suicidality were conducted based on the Korean version 5.0.0 of Mini International Neuropsychiatric Interview (MINI) (24). Patients diagnosed with MDD based on the Diagnostic and Statistical Manual of Mental Disorders (DSM)-IV were included in the MDD group. We defined the suicidal group as those with moderate or high suicidality based on the MINI suicidality item. Exclusion criteria were: (1) MDD with psychotic features; (2) comorbid major psychiatric illnesses including bipolar disorder, schizophrenia, delusional disorder, delirium, neurocognitive disorder, intellectual disability, and other mental disorders due to another medical condition; (3) history of substance-related disorders except for tobacco-related disorders within 12 months; (4) primary neurologic illness or history of brain damage; and (5) history of major physical illness. Patients’ treatment was maintained according to standard treatment guidelines for depressive disorders including antidepressant medications and did not change during the study period. HCs did not have a history of major depressive episodes and scored seven or less on the Korean version of the Hamilton depression rating scale (HAM-D) (25), with the same exclusion criteria of the MDD group applied. The study design was approved by the Institutional Review Board of Samsung Medical Center (IRB No. 2018-04-137), and all participants provided written informed consent before study participation, in accordance with the Declaration of Helsinki.

### Psychological Measures

At baseline, participants were evaluated using psychological scales including the MINI, HAM-D, the Korean version of the Hamilton anxiety rating scale (HAM-A) (26), the Korean version of the Barratt impulsiveness scale-11 (BIS-11) (27), and Clinical global impression scale - severity (CGI-S) (28).

Suicidality was evaluated based on the MINI suicidality item, which was scored according to responses to questions involving self-harm, suicidal ideation, suicidal plan, and suicide attempt. The range of suicidality scores was 0–33, and a score of 1–5 was rated as low risk, 6–9 as moderate risk, and ≥10 as high risk.

### Functional Near-Infrared Spectroscopy and Verbal Fluency Task

**FIGURE 1:**
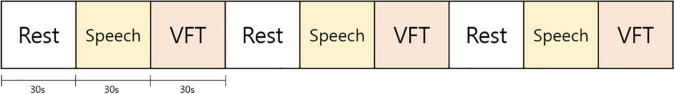
Protocol of verbal fluency test (VFT). At each visit, subjects were exposed to three consecutively repeated trials of rest, speech, and VFT. Each trial lasted 30 s. In each speech trial, subjects were asked to repeat the articulation of “giyeog-nieun-digeud-lieul” for 30 s. In the VFT trial, subjects were asked to produce as many words beginning with a specific consonant as possible.

Participants visited the experimental site 3 times at 4-week intervals, and we repeated fNIRS monitoring at each visit. At the monitoring, we extracted the concentrations of oxy-Hb using three consecutively repeated trials of rest, speech, and VFT (Figure 1), and the measured values during each trial reflected the prefrontal activity during baseline, speaking, and cognitive executive status, respectively. Each trial lasted 30 s. In each speech trial, participants were asked to repeat the articulation of “giyeog-nieun-digeud-lieul” (pronunciation of the Korean alphabets) for 30 s. During the VFT trial, participants were asked to generate as many words beginning with a specific consonant as possible. We adopted VFT to efficiently induce cognitive execution within a set time in the protocol. VFT, one of the brief psychological tests for cognitive assessment, is known to be particularly sensitive to depression due to the overlap between cognitive demands of VFT and cognitive deficits occurring in patients with depression (e.g., initiation, maintaining attention, retrieval, and persistence) (29, 30), and a previous fNIRS study reported that hemodynamic changes induced during cognitive tasks were specific to VFT, while other cognitive tasks including Stroop and the two-back task did not lead to significant changes (23).

In each channel, we calculated the average changes in oxy-Hb concentration during each VFT trial from the last 5 s of the preceding speech trial to measure the prefrontal activation corresponding to cognitive execution after excluding the effect of the speech made while performing the VFT, and the prefrontal activation at each visit was measured by averaging three consecutively calculated values. To increase the accuracy of the measurement value, subjects visited a total of 3 times at 4-week intervals and the process of fNIRS monitoring was repeated at each visit. We tried to reduce the intraindividual variation with repeated measures while reducing inattention resulting from the repetition of the same task by spacing each visit.

We used a high-density fNIRS device (NIRSIT, OBELAB, Seoul, South Korea). The sensor array comprised 24 dual-wavelength laser diodes (780/850 nm) and 32 photodetectors (31). A 3 cm distance separated the laser and detector pairs at 48 sensing areas in the prefrontal cortex including the dorsolateral prefrontal cortex (DLPFC), ventrolateral prefrontal cortex (VLPFC), frontopolar cortex (FPC), and ventromedial prefrontal cortex (VMPFC), and the optical signal variation of each channel was sampled at 8.138 Hz. The hemodynamic changes in each channel during each VFT trial were extracted using the Modified Beer-Lambert Law (MBLL). After filtering detected light signals with a band-pass filter (0.005–0.1 Hz), the signal-to-noise ratio threshold was set to 30 dB based on the resting state, removing the slow drift of physiological and environmental noise.

### Statistical Analysis

We presented continuous variables as mean ± standard deviation (SD) and categorical variables as numbers and percentages. Student *t*-tests, analysis of variance (ANOVA), and chi-square tests were used to compare the differences in factors between groups. A repeated-measures ANOVA (RM-ANOVA) was used for the comparison of changes in oxy-Hb during VFT between the groups and repeated-measures analysis of covariance (RM-ANCOVA) after adjusting for sex, age, alcohol intake, smoking status, systolic blood pressure (SBP), heart rate, and years of education was also used. The ANOVA Tukey *post hoc* tests were also applied to examine whether significant differences existed between the three groups. These statistical analyses were performed using IBM SPSS Statistics Software (version 24; IBM, New York, NY, United States). We considered a *p*-value less than 0.05 as statistically significant.

Additionally, we conducted permutation tests to compare the mean changes of oxy-Hb concentration during VFT extracted over the three visits between groups. The permutation test is a non-parametric statistical method and deals with the multiple comparisons issue when the assumptions of a parametric approach are untenable in functional neuroimaging (32). In this study, since the statistical analysis was performed on multiple brain regions, a statistical inference should be considered to reduce the risk of type I error by correcting for multiple comparisons. However, the brain regions of interest are located close to each other within the prefrontal cortex and hemodynamically highly correlated with each other (i.e., independent of each other), using classical parametric statistics with standard procedures for multiple comparison correction (e.g., Bonferroni correction) has the potential to inflate false negatives. This non-parametric resampling-based approach has been suggested as an effective and intuitive option for multiple comparison problems in fNIRS research (33–35). We ran 1000 permutations in each brain region of interest through R packages (R Foundation for Statistical Computing) and the “lmPerm” package was used for the analyses.

## Results

### Demographic and Other Characteristics

Six of the enrolled subjects dropped out from the study (1 MDD without suicidality; 2 MDD with suicidality; 3 HCs). Four subjects were excluded from the analyses due to data error (3 MDD without suicidality; 1 HC). A total of 109 subjects were included in the analyses (31 subjects of MDD without suicidality; 23 subjects of MDD and suicidality; 55 HCs) (Supplementary Figure 1). Demographic and other baseline characteristics of participants are presented in Table 1. Male subjects included 87.1% of the MDD without suicidality group, 69.6% of the MDD and suicidality group, and 49.1% of the HCs. The mean ages of the MDD without suicidality group, the MDD with suicidality group, and the HCs were 24.26 (SD, 4.46), 24.43 (SD, 4.74), and 27.38 (SD, 3.48), respectively. 96.8% of MDD without suicidality group and 95.7% of MDD with suicidality group were taking antidepressants, and none of the HCs was under psychiatric medications. Alcohol intake was reported in about 74% of subjects in the MDD groups and 96.4% of the HCs. Heavy smokers included 41.9% of the MDD without suicidality group, 26.1% of the MDD with suicidality group, and 1.8% of the HCs. The mean score of HAM-D for the MDD without suicidality, the MDD with suicidality, and the HCs were 16.29 (SD, 5.31), 18.30 (SD, 5.60), and 1.84 (SD, 1.95), respectively. The mean scores of HAM-A for the MDD without suicidality, the MDD with suicidality, and the HCs were 17.19 (SD, 7.04), 17.57 (SD, 7.51), and 2.42 (SD, 2.34), respectively. The mean score of CGI-S for the MDD without suicidality, the MDD with suicidality, and the HCs were 3.45 (SD, 0.74), 4.15 (SD, 1.26), and 1.00 (SD, 0), respectively. BIS-11 was the highest at 71.43 (SD, 14.48) in the MDD and suicidality group and the lowest in the HCs at 57.49 (SD, 9.49).

**TABLE 1:** Baseline characteristics of participants.

	MDD without suicidality (*n* = 31)	MDD with suicidality (*n* = 23)	Healthy controls (*n* = 55)	*P*-value
				
	N (%)	N (%)	N (%)	
Sex				0.002
Male	27 (87.1)	16 (69.6)	27 (49.1)	
Female	4 (12.9)	7 (30.4)	28 (50.9)	
Education				<0.001
High school	21 (67.7)	16 (69.6)	9 (16.4)	
College	7 (22.6)	7 (30.4)	33 (60.0)	
Graduate school	2 (6.5)	0 (0)	13 (23.6)	
Marital status				0.114
Unmarried	29 (93.5)	21 (91.3)	45 (81.8)	
Married	1 (3.2)	2 (8.7)	10 (18.2)	
Occupation				0.121
Permanent employee	2 (6.9)	3 (13.0)	16 (29.1)	
Temporary employee	3 (10.3)	3 (13.0)	10 (18.2)	
Housewife	0 (0)		2 (3.6)	
Student	19 (65.5)	15 (65.2)	22 (40.0)	
Unemployed	5 (17.2)	2 (8.7)	5 (9.1)	
Alcohol intake	23 (74.2)	17 (73.9)	53 (96.4)	0.004
Smoking status				0.046
No	13 (41.9)	14 (60.9)	39 (70.9)	
Ever	5 (16.1)	3 (13.0)	15 (27.3)	
Heavy	13 (41.9)	6 (26.1)	1 (1.8)	
Use of psychiatric medications				
Antidepressants	30 (96.8)	22 (95.7)	0	<0.001
Benzodiazepines	12 (38.7)	7 (30.4)	0	<0.001
Psychiatric family history	7 (22.6)	6 (26.1)	2 (3.6)	0.008
Functional impairment				<0.001
No	4 (14.3)	2 (9.1)	32 (58.2)	
Mild	14 (50.0)	7 (31.8)	7 (12.7)	
Moderate	6 (21.4)	7 (31.8)	2 (3.6)	
Severe	4 (14.3)	6 (27.3)	0 (0)	
Childhood trauma	14 (48.3)	19 (82.6)	10 (18.2)	<0.001

	**Mean (SD)**	**Mean (SD)**	**Mean (SD)**	***P-value***

Age (years)	24.26 (4.46)	24.43 (4.74)	27.38 (3.48)	0.001
Height (cm)	173.25 (7.61)	171.58 (8.31)	168.19 (7.70)	0.013
Weight (kg)	68.63 (12.21)	74.24 (18.03)	64.40 (14.31)	0.026
Systolic blood pressure (mmHg)	125.29 (14.34)	127.30 (14.83)	118.76 (15.14)	0.034
Diastolic blood pressure (mmHg)	74.48 (8.33)	75.74 (10.20)	70.62 (9.33)	0.045
Heart rate (bpm)	82.00 (10.89)	84.26 (13.00)	75.22 (8.27)	<0.001
Body temperature (°C)	36.75 (0.13)	36.71 (0.11)	36.74 (0.20)	0.636
Score of suicidality	2.71 (4.46)	10.60 (7.26)	0.07 (0.52)	<0.001
HAM-D	16.29 (5.31)	18.30 (5.60)	1.84 (1.95)	<0.001
HAM-A	17.19 (7.04)	17.57 (7.51)	2.42 (2.34)	<0.001
CGI-S	3.45 (0.74)	4.15 (1.26)	1.00 (0)	<0.001
BIS-11	69.55 (12.70)	71.43 (14.48)	57.49 (9.49)	<0.001

*MDD, major depressive disorder; HAM-D, Hamilton depression rating scale; HAM-A, Hamilton anxiety rating scale; CGI-S, Clinical global impression scale-severity; BIS-11, Barratt impulsiveness scale-11.*

### Changes in oxy-Hb Concentration During Verbal Fluency Test

**FIGURE 2:**
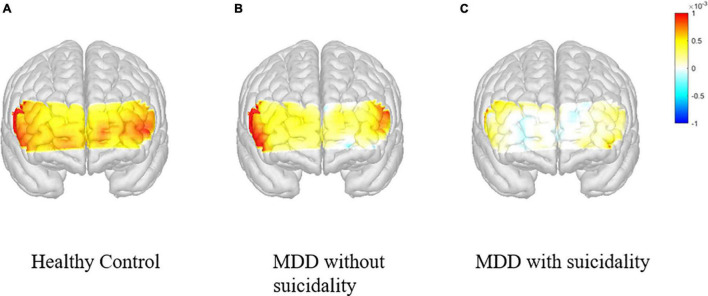
Changes in the concentration of oxygenated hemoglobin in the prefrontal cortex during VFT according to participant groups. VFT, verbal fluency test. **(A)** Healthy control. **(B)** MDD without suicidality. **(C)** MDD with suicidality.

Table 2 and Figure 2 show changes in oxy-Hb during VFT between groups. Using RM-ANOVA, significant differences were found between groups in the left FPC [*F*_(2,89)_ = 3.596, *p* = 0.031, partial η^2^ = 0.08]. When the permutation test was performed on the difference in mean values in each group, significance was also maintained (*p* = 0.013). When the groups were compared after adjusting for age, sex, SBP, HR, alcohol intake, smoking status, and years of education, there was a significant difference in the left FPC [*F*_(2,81)_ = 5.009, *p* = 0.009, partial η^2^ = 0.11]. The results of *post hoc* analyses are presented in Supplementary Table 1. The results of the comparison of changes in deoxygenated hemoglobin according to groups are presented in Supplementary Table 2.

**TABLE 2:** Comparison of changes in oxygenated hemoglobin during verbal fluency test according to groups.

(Unit: μ mol)	MDD without suicidality (*n* = 31)		MDD with suicidality (*n* = 23)		Healthy controls (*n* = 55)		RM-ANOVA			RM-ANCOVA^a^			Permutation test
						
	Mean	SD	Mean	SD	Mean	SD	F	*p*	*p* for interaction^b^	F	*p*	*p* for interaction^b^	*p*
Total prefrontal cortex	0.491	1.071	0.167	0.847	0.726	0.894	1.780	0.175	0.233	2.152	0.123	0.250	0.012
Right prefrontal cortex	0.687	1.317	0.150	1.010	0.793	1.019	1.471	0.235	0.511	1.775	0.176	0.335	0.015
Left prefrontal cortex	0.295	0.895	0.185	0.803	0.658	0.825	2.466	0.091	0.136	2.891	0.061	0.473	0.015
Right DLPFC	0.703	1.641	0.303	1.265	0.669	1.051	0.528	0.592	0.486	0.742	0.479	0.586	0.843
Right VLPFC	1.392	1.905	0.333	1.728	1.304	1.506	1.948	0.149	0.597	2.542	0.085	0.071	0.075
Right FPC	0.412	1.133	−0.019	0.809	0.586	0.900	2.180	0.119	0.373	2.385	0.099	0.255	0.005
Right VMPFC	0.366	1.177	−0.043	1.087	0.716	1.061	2.069	0.132	0.661	2.786	0.068	0.791	0.015
Left DLPFC	0.369	1.094	0.325	1.094	0.538	0.734	0.828	0.440	0.184	1.501	0.229	0.646	0.222
Left VLPFC	0.751	1.111	0.253	0.940	0.938	1.121	2.687	0.074	0.405	2.086	0.131	0.825	0.009
Left FPC	0.324	1.200	−0.007	0.768	0.662	0.963	3.596	0.031	0.532	5.009	0.009	0.650	0.013
Left VMPFC	−0.125	1.138	0.266	0.824	0.459	1.040	1.995	0.142	0.402	1.954	0.148	0.431	0.111

*MDD, major depressive disorder; DLPFC, dorsolateral prefrontal cortex; VLPFC, ventrolateral prefrontal cortex; FPC, frontopolar cortex; VMPFC, ventromedial prefrontal cortex.*

*^a^Adjusted by age, sex, systolic blood pressure, heart rate, alcohol intake, smoking status, and years of education.*

*^b^p-values for interactions between time (visit) and group.*

Supplementary Table 3 shows the comparison between HCs and MDD (with or without suicidality). After adjusting for age, sex, SBP, HR, alcohol intake, smoking status and years of education, there were significant differences between groups in the left prefrontal cortex FPC [*F*_(1,82)_ = 5.829, *p* = 0.018, partial η^2^ = 0.07], right VMPFC [*F*_(1,82)_ = 5.548, *p* = 0.021, partial η^2^ = 0.06] and left FPC [*F*_(1,82)_ = 9.997, *p* = 0.002, partial η^2^ = 0.11].

Table 3 presents the comparison between MDD patients with and without suicidality. In all prefrontal regions, changes in oxy-Hb were smaller among MDD patients with suicidality compared with those without suicidality. After adjusting for covariates, there was a significant difference in the right VLPFC [*F*_(1,35)_ = 5.028, *p* = 0.031, partial η^2^ = 0.13].

**TABLE 3:** Comparison of changes in oxygenated hemoglobin during verbal fluency test between patients with MDD and suicidality and those without suicidality.

(Unit: μ mol)	MDD without suicidality (*n* = 31)		MDD with suicidality (*n* = 23)		RM-ANOVA			RM-ANCOVA^a^			Permutation test
					
	Mean	SD	Mean	SD	F	*p*	*p* for interaction^b^	F	*p*	*p* for interaction^b^	*p*
Total prefrontal cortex	0.491	1.071	0.167	0.847	0.966	0.331	0.342	0.823	0.371	0.408	0.172
Right prefrontal cortex	0.687	1.317	0.150	1.010	1.544	0.221	0.669	1.623	0.211	0.523	0.090
Left prefrontal cortex	0.295	0.895	0.185	0.803	0.237	0.629	0.092	0.055	0.816	0.333	0.615
Right DLPFC	0.703	1.641	0.303	1.265	0.714	0.403	0.508	0.864	0.359	0.526	0.196
Right VLPFC	1.392	1.905	0.333	1.728	2.901	0.096	0.922	5.028	0.031	0.808	0.054
Right FPC	0.412	1.133	−0.019	0.809	1.687	0.201	0.473	1.221	0.277	0.233	0.140
Right VMPFC	0.366	1.177	−0.043	1.087	0.257	0.615	0.425	0.035	0.854	0.546	0.290
Left DLPFC	0.369	1.094	0.325	1.094	0.425	0.518	0.151	0.075	0.786	0.543	0.882
Left VLPFC	0.751	1.111	0.253	0.940	2.266	0.140	0.454	1.345	0.254	0.930	0.084
Left FPC	0.324	1.200	−0.007	0.768	0.937	0.338	0.328	0.521	0.475	0.352	0.154
Left VMPFC	−0.125	1.138	0.266	0.824	1.935	0.171	0.359	2.448	0.127	0.293	0.293

*MDD, major depressive disorder; DLPFC, dorsolateral prefrontal cortex; VLPFC, ventrolateral prefrontal cortex; FPC, frontopolar cortex; VMPFC, ventromedial prefrontal cortex.*

*^a^Adjusted by age, sex, systolic blood pressure, heart rate, alcohol intake, smoking status, and years of education.*

*^b^p-values for interactions between time (visit) and group.*

The time series on average changes in oxy-Hb and deoxygenated Hb concentration during VFT are presented in Supplementary Figure 2.

## Discussion

In this study, we investigated the role of fNIRS as a biomarker to diagnose and evaluate depression and suicidality in a young adult population by measuring changes in oxy-Hb of the prefrontal area during cognitive tasks. The major findings were: (1) During the cognitive task, prefrontal activation was lower in patients with MDD than in HCs. (2) Impaired prefrontal activation was more prominent in MDD patients with suicidality than in those without suicidality. (3) The differences between the three groups were significant in the left frontopolar cortex after adjusting the covariates. These findings suggest that fNIRS may be a useful tool to diagnose and characterize patients with MDD.

We found that prefrontal activation was impaired during cognitive tasks in patients with MDD compared with HCs, which is consistent with the results of previous studies (15, 16). Left frontal inactivation in patients with MDD has also been consistently reported in studies using PET or electroencephalogram (36–39). In our study, a significant difference was detected in the left FPC. The FPC is known to play a role in higher and more complex cognitive functions such as planning, problem-solving, reasoning, and episodic memory retrieval (40, 41). Combined with the findings of previous studies, our results suggest that fNIRS can be used in the young adult population to diagnose or characterize MDD.

Additionally, impaired prefrontal activation was more prominent in patients with MDD and suicidality than in those without suicidality, and this result was consistent with those of previous studies (21, 22). In our study, although statistical significance was found only in the right VLPFC, the changes in oxy-Hb involving all prefrontal regions were smaller in those with suicidality. Previous studies investigating MDD have reported the association between prefrontal function and executive impairment in patients with suicidality (42, 43). Combining these findings, our results show that executive impairment, which is a characteristic of suicidality in patients with MDD, can be correlated with the hemodynamic changes during cognitive execution and suggest that the measurement through fNIRS can be a biomarker characterizing or predicting suicidality in the young adult patients with MDD.

To our knowledge, this is the first study to identify the clinical and prognostic value of fNIRS by comparing the changes in oxy-Hb between HCs, patients with MDD without, and with suicidality among the young adult population. This study has strength in that the fNIRS data were repeatedly measured three times at intervals of 4 weeks to determine differences between groups. As a result, we tried to increase the accuracy by minimizing individual variation or error.

The study also has a few limitations. First, because we used only VFT to induce cognitive execution, the results should be interpreted within the limits of this methodology. Second, although the main outcomes were presented using RM-ANOVA and RM-ANCOVA based on the fNIRS results of three visits, the information regarding temporal variation is limited. In this study, subjects with MDD did not undergo any additional intervention during the study period and only maintained their treatment according to standard clinical guidelines, and the time by group interactions were not significant for the change of oxy-Hb concentration in the brain regions of interest. The time by group interactions was not significant. However, further studies are needed to elucidate the temporal changes in brain activity according to repetitive cognitive tasks. Third, subjects’ handedness, one of the widely investigated forms of hemispheric asymmetries (44), was not considered in the enrollment and analyses in this study. Fourth, matching for major variables such as age and sex was not conducted in subjects’ enrollment, which resulted in differences between groups, needing consideration in interpreting the results.

In conclusion, in this fNIRS study involving a young adult population, we found that, compared with HCs, patients with MDD showed decreased activation in the prefrontal cortex during cognitive tasks. In addition, patients with MDD and suicidality showed lower activation of the prefrontal cortex than those without suicidality.

## Data Availability Statement

The original contributions presented in this study are included in the article/Supplementary Material, further inquiries can be directed to the corresponding author.

## Ethics Statement

The studies involving human participants were reviewed and approved by Samsung Medical Center. The patients/participants provided their written informed consent to participate in this study.

## Author Contributions

HK: conceptualization, formal analysis, and writing—original draft, review and editing. JC: data curation and writing—review and editing. BJ, MF, DM, MP, and HSK: writing—review and editing. HJ: conceptualization, project administration, supervision, and writing—review and editing. All authors contributed to the article and approved the submitted version.

## Conflict of Interest

DM has received research support from Nordic Naturals and heckel medizintechnik GmbH. He has received honoraria for speaking from the Massachusetts General Hospital Psychiatry Academy. He also works with the MGH Clinical Trials Network and Institute (CTNI), which has received research funding from multiple pharmaceutical companies and NIMH. JC was employed by OBELAB Inc. The remaining authors declare that the research was conducted in the absence of any commercial or financial relationships that could be construed as a potential conflict of interest.

## Publisher’s Note

All claims expressed in this article are solely those of the authors and do not necessarily represent those of their affiliated organizations, or those of the publisher, the editors and the reviewers. Any product that may be evaluated in this article, or claim that may be made by its manufacturer, is not guaranteed or endorsed by the publisher.
