# Supplementary material for: Impaired Oxygenation of the Prefrontal Cortex During Verbal Fluency Task in Young Adults With Major Depressive Disorder and Suicidality: A Functional Near-Infrared Spectroscopy Study
Source: Front Psychiatry. 2022 Jun 23;13:915425. doi: 10.3389/fpsyt.2022.915425 (PMC9260011; doi:10.3389/fpsyt.2022.915425)
Supplement: Supplementary file 1 [file Table_1.docx]

**Supplementary Table 1. Posthoc analyses (Tukey method)**

| (Unit: µmol) | Group (A) | Group (B) | (A)-(B) | *SD* | *P-value* |
| --- | --- | --- | --- | --- | --- |
| Total prefrontal lobe | HC | DP | 0.235 | 0.211 | 0.508 |
|  |  | SU | 0.558 | 0.233 | 0.048 |
|  | DP | SU | 0.324 | 0.258 | 0.425 |
| Right prefrontal lobe | HC | DP | 0.106 | 0.249 | 0.906 |
|  |  | SU | 0.642 | 0.276 | 0.056 |
|  | DP | SU | 0.537 | 0.306 | 0.189 |
| Left prefrontal lobe | HC | DP | 0.363 | 0.189 | 0.137 |
|  |  | SU | 0.474 | 0.209 | 0.065 |
|  | DP | SU | 0.110 | 0.231 | 0.883 |
| Right DLPFC | HC | DP | -0.033 | 0.289 | 0.993 |
|  |  | SU | 0.366 | 0.320 | 0.488 |
|  | DP | SU | 0.399 | 0.354 | 0.499 |
| Right VLPFC | HC | DP | -0.088 | 0.376 | 0.971 |
|  |  | SU | 0.971 | 0.416 | 0.055 |
|  | DP | SU | 1.059 | 0.461 | 0.060 |
| Right FPC | HC | DP | 0.174 | 0.214 | 0.697 |
|  |  | SU | 0.605 | 0.237 | 0.032 |
|  | DP | SU | 0.431 | 0.263 | 0.234 |
| Right VMPFC | HC | DP | 0.349 | 0.247 | 0.338 |
|  |  | SU | 0.758 | 0.273 | 0.018 |
|  | DP | SU | 0.409 | 0.303 | 0.370 |
| Left DLPFC | HC | DP | 0.169 | 0.208 | 0.697 |
|  |  | SU | 0.213 | 0.230 | 0.626 |
|  | DP | SU | 0.044 | 0.255 | 0.984 |
| Left VLPFC | HC | DP | 0.187 | 0.243 | 0.723 |
|  |  | SU | 0.685 | 0.269 | 0.033 |
|  | DP | SU | 0.498 | 0.298 | 0.221 |
| Left FPC | HC | DP | 0.338 | 0.225 | 0.293 |
|  |  | SU | 0.669 | 0.249 | 0.022 |
|  | DP | SU | 0.331 | 0.275 | 0.455 |
| Left VMPFC | HC | DP | 0.585 | 0.231 | 0.034 |
|  |  | SU | 0.194 | 0.255 | 0.730 |
|  | DP | SU | -0.391 | 0.283 | 0.354 |

HC, Healthy control; DP, Major depressive disorder without suicidality; SU, Major depressive disorder with suicidality; DLPFC, dorsolateral prefrontal cortex; VLPFC, ventrolateral prefrontal cortex; FPC, frontopolar cortex; VMPFC, ventromedial prefrontal cortex

**Supplementary Table 2. Comparison of changes in deoxygenated hemoglobin during verbal fluency test according to groups**

| (Unit: µmol) | MDD without suicidality  (n=31) | | MDD with suicidality  (n=23) | | Healthy controls  (n=55) | | RM-ANOVA | |  | RM-ANCOVA ^a^ | |  |
| --- | --- | --- | --- | --- | --- | --- | --- | --- | --- | --- | --- | --- |
|  | **Mean** | **SD** | **Mean** | **SD** | **Mean** | **SD** | **F** | ***p*** | ***p* for interaction^b^** | **F** | ***p*** | ***p* for interaction^b^** |
| Total prefrontal cortex | -0.256 | 0.434 | -0.120 | 0.505 | -0.112 | 0.348 | 0.827 | 0.483 | 0.653 | 0.447 | 0.720 | 0.217 |
| Right prefrontal cortex | -0.311 | 0.607 | -0.185 | 0.695 | -0.139 | 0.548 | 0.562 | 0.641 | 0.844 | 0.174 | 0.914 | 0.365 |
| Left prefrontal cortex | -0.202 | 0.336 | -0.055 | 0.456 | -0.086 | 0.268 | 1.164 | 0.328 | 0.442 | 1.389 | 0.252 | 0.342 |
| Right DLPFC | -0.282 | 1.124 | -0.092 | 1.442 | -0.181 | 0.842 | 0.306 | 0.821 | 0.735 | 0.049 | 0.986 | 0.576 |
| Right VLPFC | -0.634 | 1.117 | -0.412 | 1.283 | -0.241 | 1.609 | 0.540 | 0.656 | 0.422 | 0.466 | 0.707 | 0.034 |
| Right FPC | -0.178 | 0.320 | -0.133 | 0.287 | -0.052 | 0.350 | 0.855 | 0.468 | 0.036 | 1.242 | 0.300 | 0.042 |
| Right VMPFC | -0.191 | 0.328 | -0.100 | 0.428 | 0.005 | 0.737 | 0.692 | 0.175 | 0.308 | 0.949 | 0.421 | 0.436 |
| Left DLPFC | -0.194 | 0.384 | -0.076 | 0.666 | -0.128 | 0.392 | 0.238 | 0.870 | 0.152 | 0.397 | 0.756 | 0.223 |
| Left VLPFC | -0.371 | 0.705 | -0.232 | 0.295 | -0.067 | 0.433 | 0.937 | 0.426 | 0.557 | 0.860 | 0.466 | 0.312 |
| Left FPC | -0.186 | 0.280 | -0.128 | 0.250 | -0.081 | 0.223 | 1.026 | 0.385 | 0.686 | 2.003 | 0.120 | 0.686 |
| Left VMPFC | -0.012 | 0.658 | 0.132 | 0.894 | -0.102 | 0.456 | 0.274 | 0.844 | 0.579 | 0.706 | 0.551 | 0.770 |

MDD, major depressive disorder; DLPFC, dorsolateral prefrontal cortex; VLPFC, ventrolateral prefrontal cortex; FPC, frontopolar cortex; VMPFC, ventromedial prefrontal cortex

^a^Adjusted by age, sex, systolic blood pressure, heart rate, alcohol intake, smoking status, and years of education

^b^*p*-values for interactions between time (visit) and group

**Supplementary Table 3. Comparison of changes in oxygenated Hb during VFT between healthy controls and patients with MDD**

| (Unit: µmol) | MDD  (n=54) | | Healthy controls  (n=55) | | RM-ANOVA | | | RM-ANCOVA^a^ | | | Permutation test |
| --- | --- | --- | --- | --- | --- | --- | --- | --- | --- | --- | --- |
|  | **Mean** | **SD** | **Mean** | **SD** | **F** | ***p*** | ***p* for interaction^b^** | **F** | ***p*** | ***p* for interaction^b^** | ***p*** |
| Total prefrontal cortex | 0.353 | 0.986 | 0.726 | 0.894 | 2.586 | 0.111 | 0.228 | 3.848 | 0.053 | 0.192 | 0.020 |
| Right prefrontal cortex | 0.458 | 1.216 | 0.793 | 1.019 | 1.143 | 0.288 | 0.346 | 1.893 | 0.173 | 0.198 | 0.152 |
| Left prefrontal cortex | 0.248 | 0.851 | 0.658 | 0.825 | 4.766 | 0.032 | 0.266 | 5.829 | 0.018 | 0.671 | 0.012 |
| Right DLPFC | 0.533 | 1.493 | 0.669 | 1.051 | 0.068 | 0.794 | 0.535 | 0.408 | 0.525 | 0.533 | 0.882 |
| Right VLPFC | 0.941 | 1.890 | 1.304 | 1.506 | 0.317 | 0.575 | 0.277 | 0.308 | 0.580 | 0.014 | 0.141 |
| Right FPC | 0.228 | 1.022 | 0.586 | 0.900 | 2.663 | 0.106 | 0.316 | 3.846 | 0.053 | 0.311 | 0.023 |
| Right VMPFC | 0.192 | 1.148 | 0.716 | 1.061 | 3.907 | 0.051 | 0.889 | 5.548 | 0.021 | 0.956 | 0.012 |
| Left DLPFC | 0.350 | 1.084 | 0.538 | 0.734 | 1.143 | 0.288 | 0.559 | 2.945 | 0.090 | 0.987 | 0.349 |
| Left VLPFC | 0.539 | 1.062 | 0.938 | 1.121 | 3.635 | 0.060 | 0.241 | 2.812 | 0.097 | 0.505 | 0.090 |
| Left FPC | 0.183 | 1.042 | 0.662 | 0.963 | 6.558 | 0.012 | 0.446 | 9.997 | 0.002 | 0.790 | 0.013 |
| Left VMPFC | 0.041 | 1.026 | 0.459 | 1.040 | 2.048 | 0.156 | 0.476 | 1.690 | 0.197 | 0.625 | 0.028 |

MDD, major depressive disorder; DLPFC, dorsolateral prefrontal cortex; VLPFC, ventrolateral prefrontal cortex; FPC, frontopolar cortex; VMPFC, ventromedial prefrontal cortex

^a^Adjusted by age, sex, systolic blood pressure, heart rate, alcohol intake, smoking status, and years of education

^b^*p*-values for interactions between time (visit) and group

**Supplementary Figure 1. Flowchart outlining subject selection**


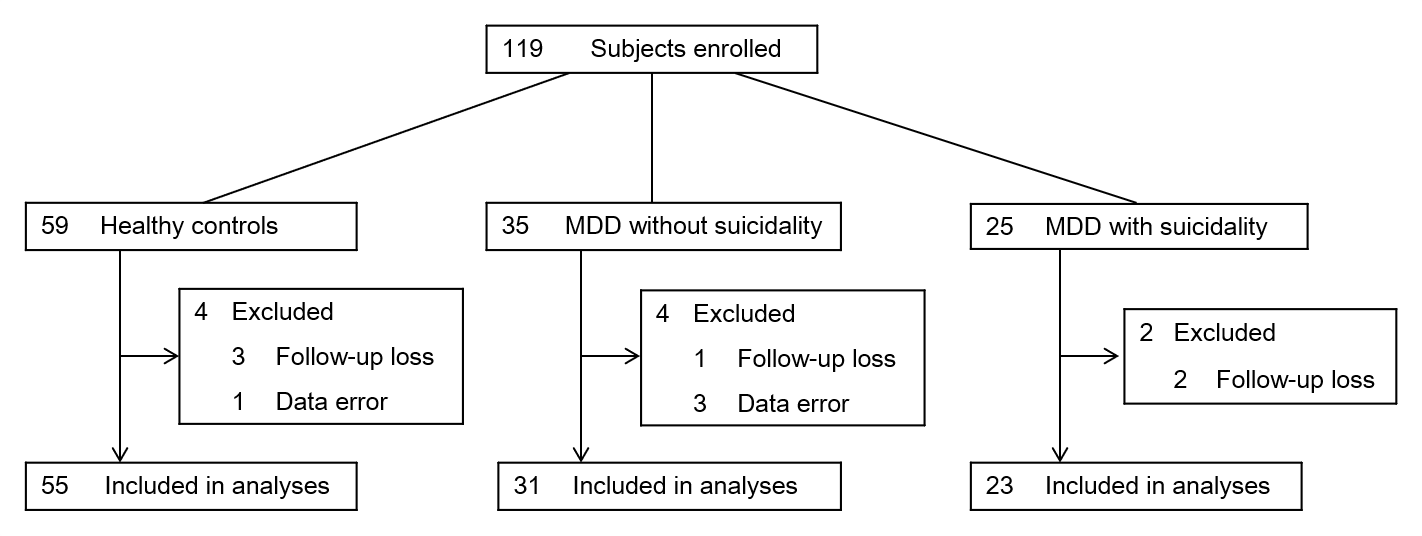


MDD, major depressive disorder

**Supplementary Figure 2. Time series on concentration of oxygenated hemoglobin and deoxygenated hemoglobin during VFT in each group**

1.
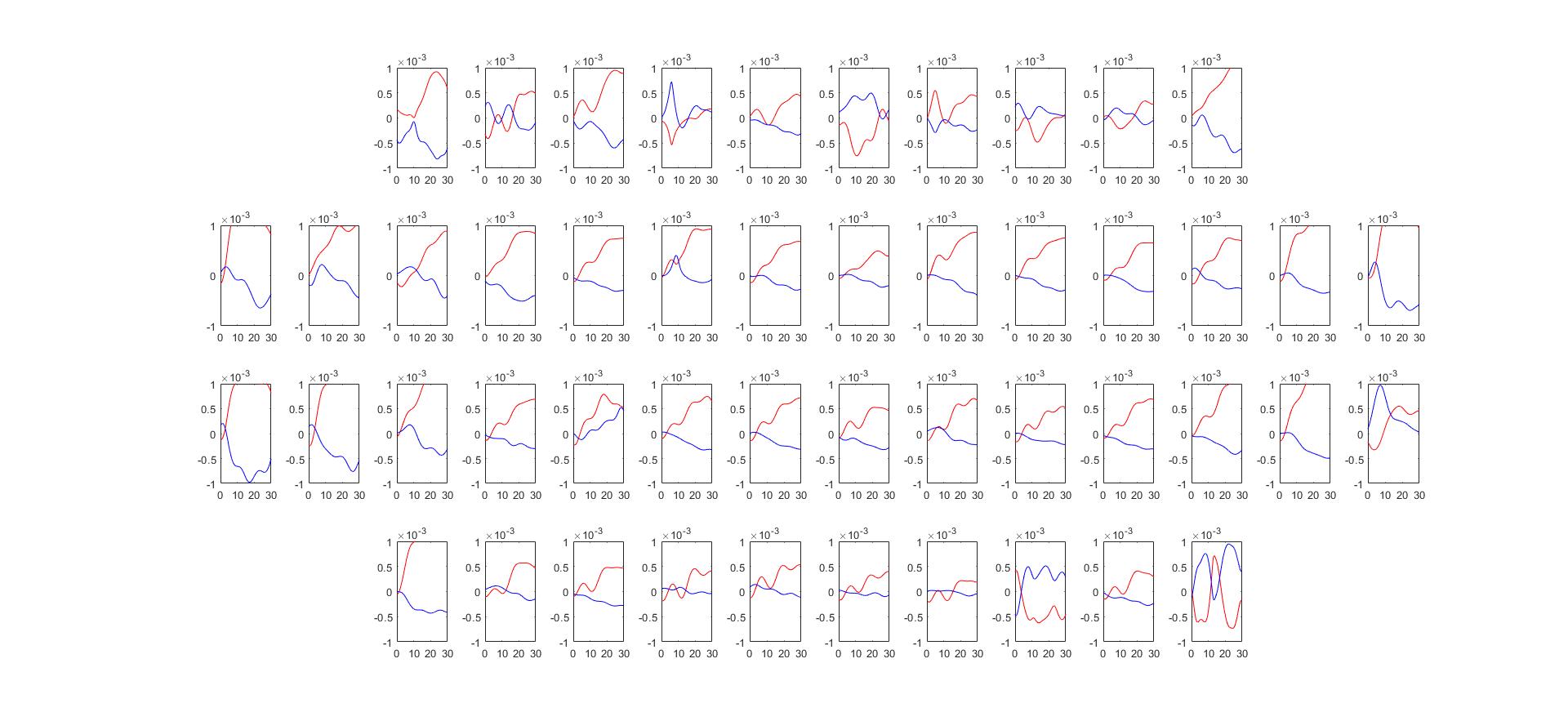
MDD without suicidality
2.
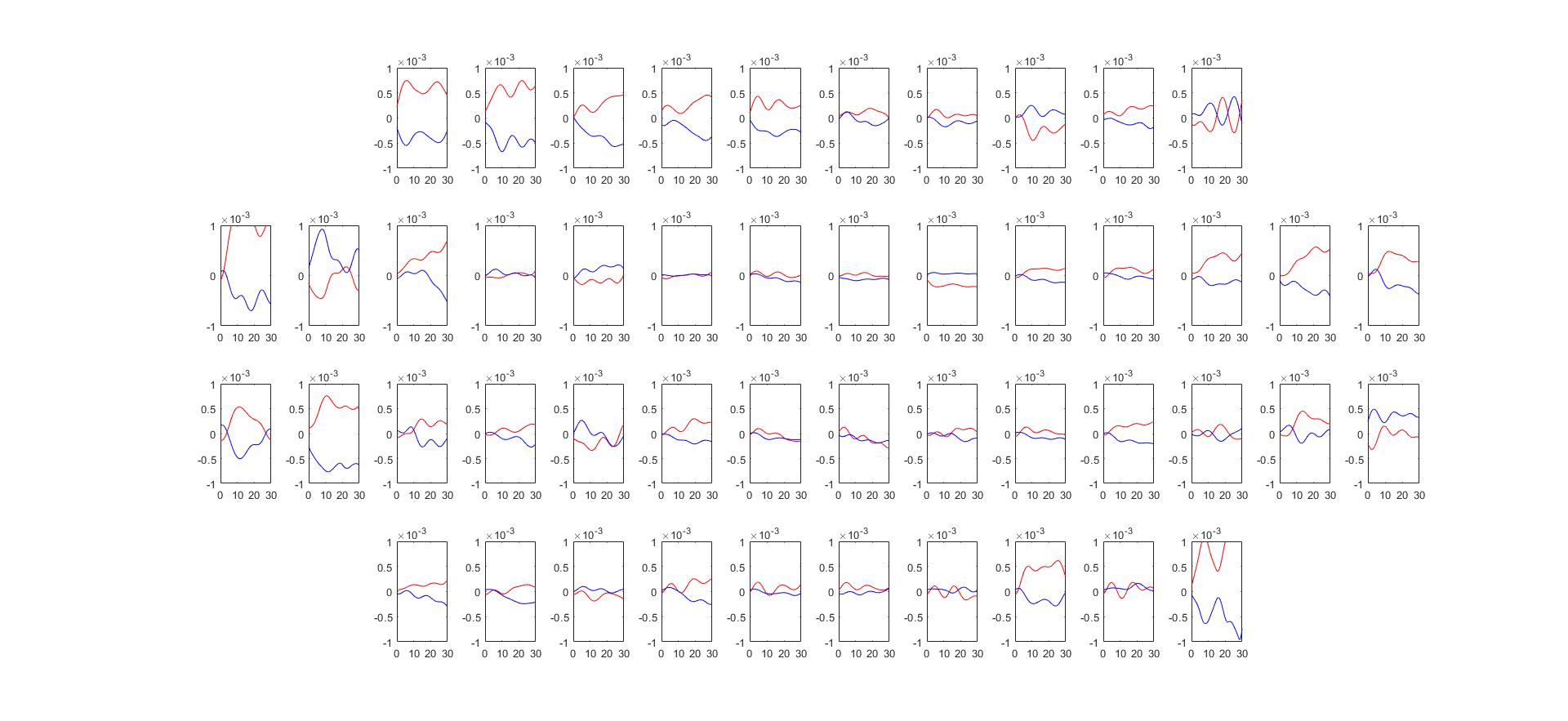
MDD with suicidality
3.
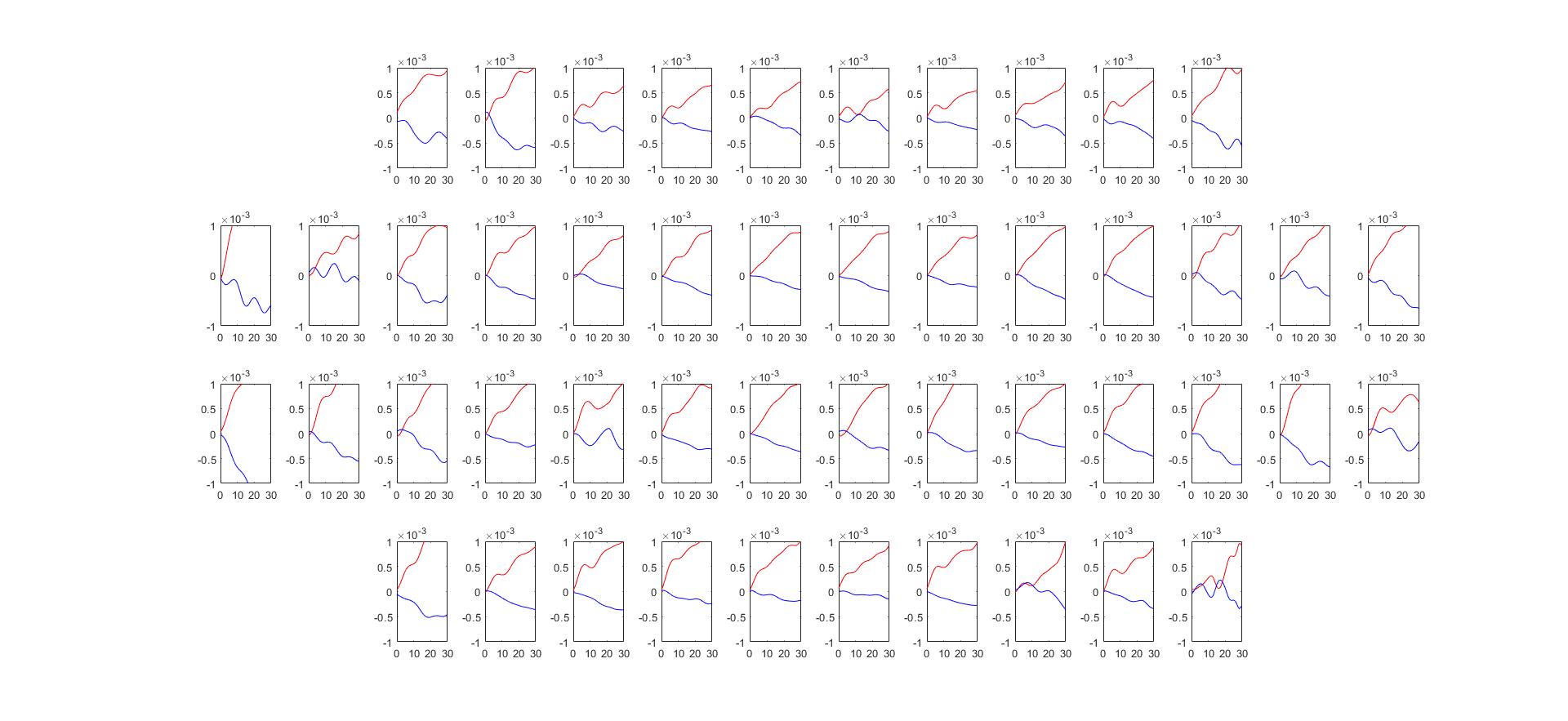
Healthy controls

MDD, major depressive disorder

Red lines indicate average changes in oxygenated hemoglobin and blue lines indicate average changes of reduced hemoglobin during the verbal fluency test
